# Conditional independence relations among biological markers may improve clinical decision as in the case of triple negative breast cancers

**DOI:** 10.1186/1471-2105-10-S12-S13

**Published:** 2009-10-15

**Authors:** Federico M Stefanini, Danila Coradini, Elia Biganzoli

**Affiliations:** 1 Dipartimento di Statistica 'G.Parenti', Università degli Studi di Firenze, viale Morgagni 59, 50134 Firenze, Italy; 2 Istituto di Statistica Medica e Biometria "G.A.Maccacaro", Università degli Studi di Milano, Italy; 3 Istituto di Statistica Medica e Biometria "G.A.Maccacaro", Università degli Studi di Milano and Istituto Nazionale dei Tumori, via Venezian 1, 20133 Milano, Italy

## Abstract

The associations existing among different biomarkers are important in clinical settings because they contribute to the characterisation of specific pathways related to the natural history of the disease, genetic and environmental determinants. Despite the availability of binary/linear (or at least monotonic) correlation indices, the full exploitation of molecular information depends on the knowledge of direct/indirect conditional independence (and eventually causal) relationships among biomarkers, and with target variables in the population of interest. In other words, that depends on inferences which are performed on the joint multivariate distribution of markers and target variables. Graphical models, such as Bayesian Networks, are well suited to this purpose. Therefore, we reconsidered a previously published case study on classical biomarkers in breast cancer, namely estrogen receptor (ER), progesterone receptor (PR), a proliferative index (Ki67/MIB-1) and to protein HER2/neu (NEU) and p53, to infer conditional independence relations existing in the joint distribution by inferring (learning) the structure of graphs entailing those relations of independence. We also examined the conditional distribution of a special molecular phenotype, called triple-negative, in which ER, PR and NEU were absent. We confirmed that ER is a key marker and we found that it was able to define subpopulations of patients characterized by different conditional independence relations among biomarkers. We also found a preliminary evidence that, given a triple-negative profile, the distribution of p53 protein is mostly supported in 'zero' and 'high' states providing useful information in selecting patients that could benefit from an adjuvant anthracyclines/alkylating agent-based chemotherapy.

## Background

Oncological patients with similar clinical features could have heterogeneous dynamics of disease recurrence and response to therapies. Such a behaviour is prevalently associated to biological features [1]. For this reason, molecular oncology is actually focused on the identification of cancer phenotypes with homogeneous biological profile that could explain the differential response of the disease to the therapies and associable to specific biomarkers. In such a perspective, according to the pharmacogenomic paradigm, biomolecular cancer staging systems have been proposed as alternative to classical systems [2]. However, the use of "omic" techniques that have initially opened potential developments it has been followed by frustrations and doubts about the real clinical usefulness of results [3]. Whereas the majority of the oncologists is still far from the application of the so-called "genomic signatures" to support clinical decision, physicians interest is focusing on the identification of subgroups of patients responsive to the pharmacological treatments better exploiting the interrelationships among different biomarkers that in a statistical perspective, are observable variable (presence/absence of a given protein, mRNA or protein level, cell morphology traits) possibly associated to at least one target (outcome) variable. Two typical questions involve biomarkers: the current patients state, referred to disease profiling or subtyping and the patients outcome (for example, the five-year horizon since surgery), referred to disease dynamics. As regard the disease profiling, a shared interest by biomedical scientists is about the associations existing among the different biomarkers that characterize specific metabolic assets of the disease according to its natural history, genetic and environmental determinants. To study such associations, the use of binary/linear (or at least monotonic) correlation measures is a general choice as the application of multivariate analysis techniques aimed to the clustering of subjects and their biological features. However, such conventional analysis tools could be limiting since binary associations, although related, provide few insights about features of the joint multivariate distribution. In fact, from an inferential point of view, the full exploitation of a biological information is based on the knowledge of all direct/indirect conditional independence relations [4] among the biomarkers analyzed and with the target variables in the population of interest. Graphical models are aimed to face such a problem and among them, Bayesian Networks (BNs) have interesting properties that allow to address the above arose questions in a full probabilistic setting. Indeed, among the leading technologies, BNs are able to describe and derive the conditional independence (CI) relations existing in highly structured stochastic systems ([5], and references therein). Besides simplifying communication among experts of different fields, BNs support causal reasoning [6] and the development of efficient algorithms for conditioning and marginalization through exact (local) computation [6]. Although the increasing hype surrounding BNs [7] partially comes from records of success in processing biomedical [8] and molecular data [9], Directed Acyclic Graphs (DAGs) are a valuable tool for reasoning on the design of a study [10]. To better understand the information provided by biomarkers, in the present paper a probabilistic interpretation of DAGs was applied in a case study previously published by Ambrogi et al. [11], although the algorithms employed to learn DAGs structure was tailored to learn causal BNs too. Such an analysis was performed to further characterize CI relations among five biomarkers: estrogen receptor (ER), progesterone receptor (PR), a proliferative index (PROLN) from the determination of the marker Ki67/MIB-1, the receptor tyrosine kinase HER2/neu (NEU) normally involved in the signal trasduction pathways leading to cell growth/differentiation and the p53 (P53) involved in cell cycle arrest and DNA repair or apoptosis. In particular, the possible heterogeneity of CI relationships for certain classes of patients was addressed by learning the structure of MultiDAGs, a generalization of DAGs developed to represent some context-specific conditional independence relationships. Finally, to challenge the tool we examined a peculiar breast cancer molecular phenotype, the so-called triple-negative group, in which ER, PR and HER2 are absent. Since, in triple-negative tumors p53 protein appears heterogeneously expressed [13], suggesting that it may be associated with specific subgroups, we investigated the distribution of p53 conditioned to such a phenotype.

## Results and discussion

### Structural learning without AGE

**Figure 1:**
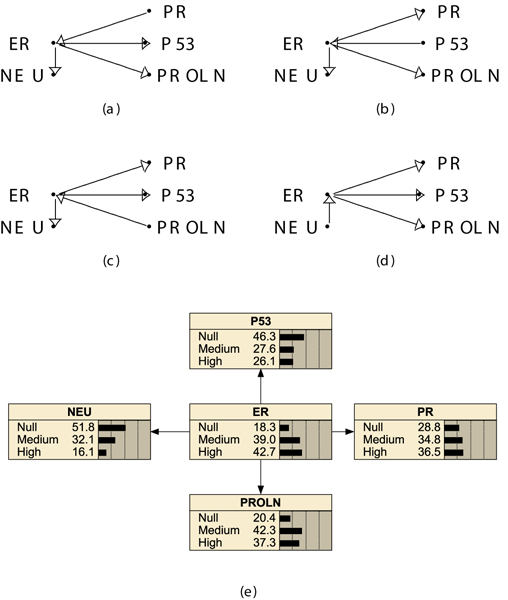
**Learned structures without AGE**. Equivalence class of DAGS obtained by search with the BDe score (5 nodes). DAG (e) is also the subDAG obtained from DAG in Figure 2 (a) by deleting AGE. Belief bars represent estimated marginal probabiliti values at each node. A more compact representation would be obtained by removing edge orientation from all arrows, although this would hide the different causal information carried by the above DAGs even if equivalent as regards CI relationships.

The first structural learning task was performed on the full set of 633 molecular profiles made by five biomarkers. Several run of a greedy search (one edge change and score) and of a simulated annealing algorithms were performed with equivalent sample size *N *equal to 9. The prior distribution on structures was taken as uniform, so that *p*(*z *| *ξ*) ∝ 1 does not make any structure more plausible a-priori. The inspection of top scored networks always confirmed the results shown in Figure 1. The selected structures are members of an equivalence class defined by the same set of conditional independence relations, a statement derived from the application of the Directed Markov Factorization (DMF) theorem. Variable ER separates all other pairs of variables, therefore pairs of variables separated by ER are conditionally independent given ER. The BDe log-score is equal to -3202.7056.

In Figure 1(e), one of the learned BNs is displayed using belief bars representing marginal probability values at each node after parameter learning based on the 633 instances of the case study.

From the learned structures we judged that pairs of variables are typically correlated despite the fact that conditioning on ER make them independent. This is a reasonable but not trivial finding, because the usual practice of inspecting the empirical distribution of pairs of variables is not prone to reveal such CI relations. From the DMF theorem applied to sets of nodes {*P*53}, {*NEU*, *PROLN*, *PR*} and {*ER*}, it follows that, for example, given ER no further information is provided by other markers on p53.

### Structural learning including AGE

It is well known that the metabolism of oestrogens and of progesterone is related to patient's AGE, even though AGE might also affect other markers as regards their conditional distribution. Therefore, the second structural learning task was performed on the full set of 633 molecular profiles composed of five biomarkers including variable AGE among nodes in *V*.

Several runs of greedy search and simulated annealing algorithms were performed with equivalent sample size equal to 9 and a uniform prior distribution for *Z*. The inspection of top scored networks allowed to find the equivalence class of structures and to inspect CI relationships. The DBe log-score is equal to -3752.4043, while the log-score of the same network with node AGE disconnected from other nodes is -3757.7314.

**Figure 2:**
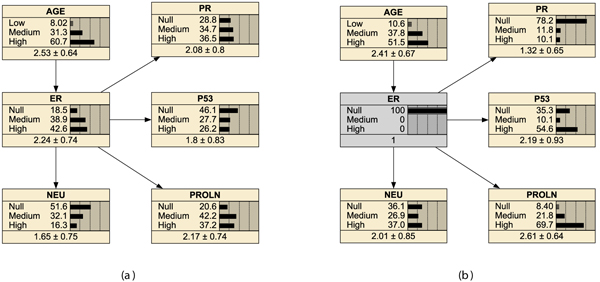
**Learning with AGE**. Marginal distributions for the selected structure including AGE (six nodes), after parameter learning (a) and conditioning on *ER *= *Null *(b).

In Figure 2 just one member of the equivalence class is shown. It was chosen by exploiting the a-priori information of molecular pathologists. All the other members (not shown) of the equivalence class are characterized by an arrow from ER to AGE. While such edge orientation is probabilistically sound it is counterintuitive on causal ground because AGE is likely to cause changes in the distribution of five markers and not vice-versa probably due to physiological variation in the biologic behaviour associated with aging (see the discussion).

### Structural learning of a multiDAG: AGE

The role played by age on estrogen receptors is well documented in the literature, therefore it is very interesting to investigate about the presence of heterogeneous CI relations within groups of patients belonging to different age classes. In other words, CI relationships among markers could be different within the three age classes.

**Figure 3:**
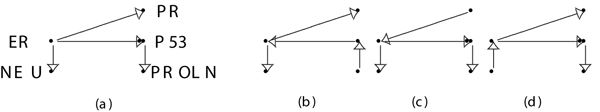
**MultiDAG with AGE = High**. Equivalence class of structures within patients with AGE equal to High. Node labels are shown only in DAG (a).

Therefore, we performed an exhaustive search by scoring structures under the BDe metric for each age class separately. The equivalent sample size *N *was always equal to 9. Within the class of low AGE the equivalence class of structures is similar to (a, b, d, e) in Figure 1 after removing edge from-to PROLN because it is not connected to any other node. Within the class of medium AGE the equivalence class of structures is equal to DAGs in Figure 1. Within the AGE class high we obtained the equivalence class of structures shown in Figure 3.

Then, we compared the best DAG against the multiDAG for the distinguished variable AGE by means of the Bayes factor (BF). The log-BF is greater than 31 therefore the best DAG is better supported by data than multiDAG AGE.

### Structural learning of a multiDAG: ER

Variable ER have been found to play a key-role in making all the other pairs of variables conditional independent. Moreover, from a theoretical point of view, the absence of estrogen receptors could modify the pattern of association among other biomarkers.

**Figure 4:**
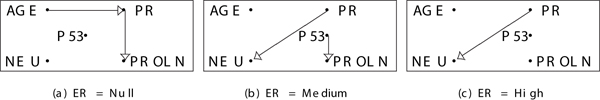
**MultiDAG for the distinguished variable ER**. DAGs belonging to equivalence class of structures for each value of the distinguished variable ER.

An exhaustive search in the space of MultiDAGs for the distinguished variable ER have been performed by scoring structures under the BDe metric for each ER class separately. The equivalent sample size *N *was always equal to 9. Results are shown in Figure 4 in which one member of the equivalence class of DAGs is shown for each value of the distinguished variable ER. Given ER, CI holds in general among biomarkers but with some exceptions. PR is not independent on AGE for ER Null, PROLN is not independent of PR given ER Null. Moreover, for ER Medium, PR is not independent on NEU and PROLN is not independent on p53. Finally, for ER High, NEU is not independent on PR.

We compared the best DAG against the multiDAG for the distinguished variable ER by calculating the Bayes factor (BF). The log-BF is greater than 6.4 therefore the multiDAG-ER model is substantially favoured as explanation of observed data.

### Triple-negative profiles

Triple-negative cancers are defined as those tumors having an ER, PR and HER2/neu-negative status. Although they account for 10 – 17% of all breast carcinomas, triple-negative cancers represent a relevant clinical issue because of the high incidence in younger patients and the higher aggressiveness than tumors pertaining to other molecular subgroups [12,13]. This aggressiveness is best illustrated by the fact that the peak risk of recurrence is between the first and third years of follow up and the majority of deaths occur in the first five years following therapy [16]. From a biological point of view, salient features of triple-negative breast cancers include overexpression of EGFR and c KIT, high proliferative rates, frequent genomic alterations, phenotypic similarity to BRCA1-associated cancers and frequent mutations of Tp53 [14,15]. In particular, p53 appears heterogeneously expressed, suggesting that it may be associated with specific subgroups. Since TP53 gene mutations are predictive of response to taxanes, p53 expression represents a useful biological marker to select, among the triple-negative tumors, those more likely to benefit from taxane versus anthracyclines/alkylating agent-based chemotherapy [16,17]. Therefore, we examined the p53 marginal distribution conditioned on the subset of triple-negative cancers included in the case series analyzed by Ambrogi et al. [11].

Bayesian parameter learning for a given structure *z *is performed by calculating the posterior distribution of *θ*^(*z*) ^given the database of cases . The expected value of *θ*^(*z*) ^is a point estimate of networks parameters, as such it may be used to perform inferential tasks like marginalization and conditioning. It is of some interest to show belief bars representing marginal distributions at each node calculated from the point estimates of :

(1)

where *V*\*v*_*i *_is the set difference made by all nodes but *v*_*i*_. Moreover, here the interest is also focused to the conditional distribution of p53 given *ER *= *Null*, *p*(*x*_*P*53 _| *x*_*ER *_= 0, , *z*, , *ξ*), which is obtained by using an exact algorithm for evidence propagation.

The multiDAG with ER as distinguished variable is the best structure to be exploited while obtaining the above mentioned point estimates. In the our case study, given *ER *= *Null*, the best DAG and the best MultiDAG-ER provide essentially the same point estimate of the distributions for *P*53, up to some rounding, so that in Figure 2(a) the marginal distribution of p53 is shown after parameter learning. The marginal distribution of p53 is for about one half on the null value (0.46) and about one quarter on the two other values each. The distribution of p53 changes by conditioning on a triplo-negative state, namely to *ER *= *Null*. Similar considerations motivate the use of Figure 2(b) to inspect the conditional probability table (CPT) of node P53 after conditioning to *ER *= *Null*. The probability of a null value for P53 decreases to 0.35 and the probability of a medium value is lowered to 0.10, while 0.54 is the probability of a *P*53 value equal to high. The estimate of the marginal distribution puts a relevant fraction of the mass, 90%, on the two extreme states, Null or High. Heterogeneous patients dynamics might depend on such pretty much substantial difference which is worth to be further characterized.

**Figure 5:**
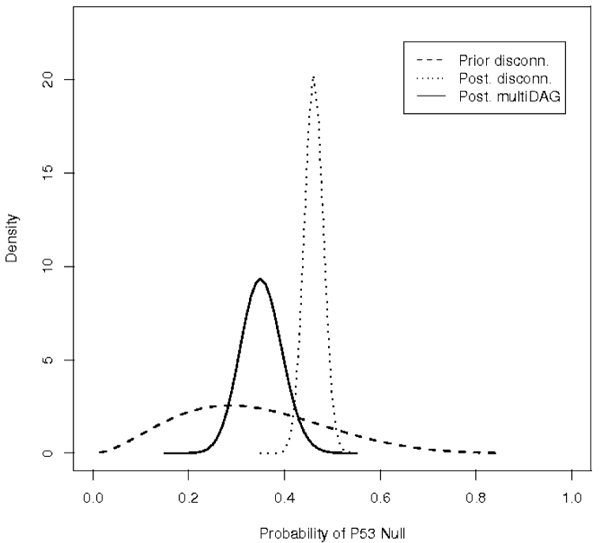
**Probability Distribution of P53**. Probability of event *P*53 = *Null *under three different circumstances: prior information with P53 disconnected (dashed line), posterior information with P53 disconnected (dotted line), posterior information given *ER *= *Null *in structure multiDAG-ER (continuous line).

A point estimate of  typically neglects the uncertainty left after learning. We address this issue for the P53 node, due to the relevance for clinical decision making, by exploring the distribution of the quantity indicating the probability of event {*P*53 = *Null*} under different models, Figure 5. The more disperse curve on the left (dashed line) refers to the prior belief for such event before learning structure and parameters when marginal independence is assumed among biomarkers, thus P53 is disconnected. The peaked curve on the right (dotted line) represents the posterior distribution of {*P*53 = *Null*} for a DAG without connections from to P53, thus P53 is disconnected. Finally, the continuous line in the middle is the final marginal distribution obtained after structural and parameter learning given a triplo-negative profile under a multiDAG ER model. The comparison reveals that the initial (prior) distribution is quite disperse while posterior distributions based on two different DAGs substantially differ. The Bayes factor is strongly in favour of the learned multiDAG structure therefore we can also estimate the bias induced by a poorly supported model, like a marginal independence model as the difference between point estimates under the two mentioned models.

### Comparison of learned structures against expert belief

Structural learning of BNs has been performed under a constant prior distribution on the space of structures. We used prior information elicited from molecular biologists about the presence of marginal association on pair of variables to evaluate the performances of the learning algorithm.

Stating the degree in a verbal way, the following associations were considered AGE-ER (strong), AGE-PR (weak), AGE-NEU (weak), ER-p53 (strong), PR-p53 (strong), ER-PR (strong), NEU-p53, NEU-PROLN (weak). By applying the separation theorem for directed graphs [6] on DAG shown in Figure 2 with an empty separating set, we checked out that all the stated marginal associations hold true in the learned structure. More formal and computational heavy approaches for structural learning are needed to exploit prior information about association in a quantitative way, for example by using an informed scoring function [18].

## Conclusion

Although the research trend in molecular phenotyping of cancer is aimed to the identification of biologic profiles related to the response to therapies, statistical approaches adopted so far in papers published on clinical journals, mainly resort to a conventional bivariate assessment of Pearson correlation coefficients among different single biomarkers. The examination of correlation matrices looking to pairs of variables, is performed implicitly assuming a joint Gaussian distribution and the appropriateness of linear associations. Moreover conditional associations are often neglected. The use of more advanced multivariate techniques for clustering and projection, has only partially supplied the need of dealing, in a more extended way, the bulk of information provided by multiple markers determinations such those arising from high-throughput genomic/proteomic assays. Such techniques are mainly set up on bivariate distances based on pairs of canonical variables, thus preventing a deeper view of high order associations among biomarkers. The use of graphical models for the study of conditional independence structures in joint multivariate distributions could represent an appropriate solution for the above issues. However, such techniques are still far from a wide diffusion, even within the biostatistical community. Within the graphical model class, Bayesian Networks (BNs) show interesting properties for the study of conditional independence (CI) relationships, addressing the above questions in a full probabilistic setting. Having their major relevance in the study of causal relationships, BNs can be also extended to account for latent unmeasured variables. In addition, the chance of heterogeneity in CI relationships for certain classes of patients can be also addressed by learning the structure of MultiDAGs. In the example considered in the present work, five conventional biomarkers and patients age were related by learning probabilistic networks. Although the central role of ER in breast cancer biology is well established, particularly as prognosticator of response to an endocrine therapy, less evident is the result of conditional independence among other markers given ER. The comparison between the best multiDAG structure and the top scored DAG revealed that inferences on p53, given a triple-negative profile, was substantially stable, and that the shift of probability mass towards extreme p53 values (low and high) unchanged. Such a novel information, initially explored with clustering and visualisation [11] and now confirmed by BN analysis could be relevant in the treatment of triple-negative patients who now appear split according to p53 values. This finding is not trivial because, if so far, the clinical class of triple-negative cancers has been assimilated to basal-like tumors (one of the five main phenotypes identified by gene expression analysis), present evidence support the emerging opinion that triple-negative and basal like breast cancers are not synonymous as the triple negative group, in addition to basal-like tumors, encompasses also normal breast like cancers [14]. Taking into consideration that normal breast-like tumors do not respond to neoadjuvant chemotherapy as well as basal-like cancers do [23], the identification by p53 of the subset of patients that could benefit from an adjuvant anthracyclines/alkylating agent-based chemotherapy is great clinical relevance.

In this work we supported a probabilistic semantic for BNs, but structural learning using the BDe score is also suited to learn causal relationships. In a causal framework the learned structures should be considered as tentative hypotheses to be further investigated in controlled experiments, possibly under randomization. While a (validated) causal network is essential to predict the effect of intervention, like a drug therapy, the quality of inferences strongly depend on the causal sufficiency of the considered variable, that is the lack of unmeasured (hidden) variables affecting two or more observed markers. Recent research has addressed the issue of estimating causal effects in presence of hidden variables but in those settings the structure is assumed known. Therefore, we plan to extend our analysis by investigating the presence of relevant hidden variables. Structural learning of CI relationships was performed after including variable AGE, known to be associated with estrogen plasma concentration and corresponding estrogen receptor levels. Within the equivalence classes of learned structures we preferred DAGs carrying the oriented edge AGE-to-ER, even if a deeper casual explanation might recognize that variable AGE per-se is deprived of a biological meaning, although related to the biological process of cancer development.

The prior distribution *p*(*z *| *ξ*) formally appearing in equation (3) was omitted during the maximization of the score because a uniform distribution on all the set of DAGs on the six variables was chosen. Some prior beliefs on the presence of marginal associations were used only to check the quality of learned structures. Nevertheless, further work should address the issue of formally eliciting expert beliefs both about associations and causal relationships [19]. Suitable heuristics might reduce the burden due to elicitation by focusing on preminent features characterizing expert's prior belief [18]. Issues still open are related to the need of discretisation of the variables considered in the BN model. Since such an operation should be based on sensible cut-off values often unavailable, every result and possible conclusions should be taken at exploratory level.

## Methods

In this section we present the case study due to Ambrogi et al. [11], then a short introduction to Bayesian Networks is provided. The Bayesian Dirichlet equivalent score is defined as the objective function to be optimized by search algorithms for structural learning. This section ends with the definition of MultiDAGs, a generalization of DAGs developed to represent some context-specific conditional independence relationships.

Original data were processed into transformed variables using R [20], which has been also used in some prototyping of calculations and displays. Much of the computation has been performed in Java  using the Eclipse platform . Our own Java code complements two commercial BN engines, and it was developed especially to extend their functionalities and to check critical steps performed by mean of software libraries.

### A case study on bioprofiles

We consider a subset made by 633 archival tissue samples originating 5 validated markers from patients who underwent surgery for primary infiltrating breast cancer between 1983 and 1992 at the University of Ferrara, Italy [11]. We also considered the variable AGE for its well known role in estrogen expression. The original study aimed to identify tumor profiles of clinical relevance based on immunohistochemical molecular markers measured within a single hospital.

The biomarkers here considered are: estrogen receptors (ER), progesterone receptors (PR), a proliferation index (PROLN = Ki-67/MIB1), and two proteins (HER2-NEU) and (p53). In the present study we also considered the variable AGE due to the important relationship between patient age, which reflects woman menopausal status, and estrogen status.

The original variables were all transformed to discrete ternary variables following the suggestions of experts in the measurement process (Table 1). The empirical marginal distributions of absolute frequencies are shown in Table 1. The dataset does not contain missing values.

**Table 1:** Aggregation of original data

	(a) Original classes						(b) Absolute frequencies in aggregated classes		
	1	2	3	4	5	6	1	2	3
PR	1	2	2	2	3	3	182	220	231
ER	1	2	2	2	3	3	116	247	270
NEU	1	2	2	2	3	3	328	203	102
PROLN	1	2	2	3	3		129	268	236
P53	1	2	3	3			293	175	165

AGE	< 50	[50, 60]	> 60				50	198	385

(a) Aggregation of original classes performed before analyzing data. The original discrete values are reported in the first row while integer numbers referring to final classe are shown in the body of the table. (b) Empirical marginal distributions of absolute frequencies after transformation of original data.

### Bayesian networks

A directed graph  is a pair (*V*, *E*), with *V *= {*v*_1_, *v*_2_,...,*v*_*K*_} a finite set of nodes which label the elements of random vector  and *E *a subset of the Cartesian product *V *× *V*. If (*v*_*i*_, *v*_*j*_) ∈ *E *then (*v*_*j*_, *v*_*i*_) ∉ *E *and the ordered pair (*v*_*i*_, *v*_*j*_) corresponds to the oriented edge *v*_*i *_→ *v*_*j*_. Only oriented edges are allowed in .

Let (, *v*_*j*_), (, *v*_*j*_),... be all the elements of *E *in which *v*_*j *_follow another node (oriented edges into *v*_*j*_), then the set of parents of node *v*_*j *_is *pa*(*v*_*j*_) = {, ,...}. If (*v*_*i*_, ), (*v*_*i*_, ),... are all the elements in which *v*_*i *_precedes another node then the set of children of node *v*_*i *_is . Two nodes are connected if an edge joins them. A path (*v*_0_,..., *v*_*k*_) is a sequence of nodes in which pairs *v*_*i*_, *v*_*i *+1 _are connected by an edge. A directed path is a path in which each edge is oriented from *v*_*i *_to *v*_*i *+1_. Set *an*(*v*_*i*_) collects the ancestors of node *v*_*i*_, that is nodes originating directed paths reaching node *v*_*i*_. All nodes of a directed path originated in *v*_*i *_are descendants of *v*_*i*_, and they are elements of set *de*(*v*_*i*_). A cycle in a directed graph is a directed path where the first and last node are equal, *v*_0 _= *v*_*k*_. A directed graph  without cycles is a Directed Acyclic Graph (DAG).

A Bayesian network (, ) is a pair made by a DAG whose nodes *v*_*i *_∈ *V *refers to a random variables hereafter discrete with range  and by a distribution Markov with respect to . A probability distribution  for random variables indexed in *V *is said to be Markov with respect to  if the joint distribution factorizes according to the DAG parents-to-children structure:

(2)

where  is a realization of the random vector made by variables whose labels belong to parents set *pa*(*v*_*i*_). The distribution associated to a node  is conditional to the set of random variables labeled by parent nodes, *pa*(*v*_*j*_).

The lack of an arrow from *v*_*i *_to *v*_*j *_means irrelevance of *v*_*i *_in predicting *v*_*j*_, that is conditional independence. Further conditional independence relations may be derived using the directed global Markov criterion on moralized DAG [6]. The moral graph  of DAG  is equal to the starting DAG but without edges orientation in which further (undirected) edges are added to join pairs of nodes sharing the same child without being originally connected. Two nodes *v*_*i *_and *v*_*j *_are separated i  by a set of nodes *S *⊂ *V*\*v*_*i*_, *v*_*j *_if all the path from *v*_*i *_and *v*_*j *_contain at least one node belonging to *S *in the moral graph . The extensions of separation of two subsets of nodes is straightforward. The Directed Markov Factorization (DMF) theorem states that given three disjoint subsets *A*, *B*, *S *of *V *random vectors *X*_*A *_and *X*_*B *_are conditionally independent given *X*_*S *_if *S *separates *A *from *B *in the moral subgraph made by *A *∪ *B *∪ *S *and their ancestral sets.

The structure of a Bayesian network is sometimes unknown and we define a variable *Z *on a subset *χ*_*Z *_of positive integers so that *Z *is one-to-one with the set of all DAGs defined on a given collection of nodes *V*.

The expert's degree of belief about the structure of a BN is represented by a conditional probability mass function *p*(*z *| *ξ*) given the considered domain context *ξ*.

In the simplest observational design, a database  of *n*_*d *_conditionally independent realizations from the distribution  are taken without missing values. Structural learning of a BN by means of a Bayesian score amounts to process the database  to infer the conditional independence relations existing in the joint distribution of the random vector. A Bayesian score is a function obtained by integrating out parameters of conditional distributions appearing in (2):

(3)

where  is the vector of parameters for structure *z*, that is parameters  for all *v*_*i *_∈ *V *are conditional probability tables (CPTs) espliciting factors *p*(|*θ*, *z*, *ξ*). The notation *θ*_*i*, *j *_= (*θ*_*i*, *j*, 1_,...,*θ*_*i*, *j*, *s*_,...) refers to the column *j *of the CPT for node *v*_*i *_and by using *s *as a row index it follows that ∑_*s *_*θ*_*i*, *j*, *s *_= 1.

Under the assumptions discussed in [21] and here retained, standard Bayesian updating formulas with conjugate families provide a closed-form expression for (3). The likelihood function *p*(|*θ*, *z*, *ξ*) is a product of multinomial probability mass functions and conjugate prior distributions for *θ*^*z*^s is defined by the product of Dirichlet probability density functions:

(4)

where *θ*_*i*, *j*, *s *_is the probability value of *X*_*i *_taking value in row *s *given parents state in column *j*, and where (*α*_*i*, *j*,1_,...,*α*_*i*, *j*, *s*_,..., ), are parameters of a Dirichlet distribution at node *v*_*i *_given parents state *j*, with *α*_*i*, *j *_= ∑_*s *_*α*_*i*, *j*, *s *_and *r*_*i *_the number of states of *X*_*i*_.

The choice of parameters (*α*_*i*, *j*,1_,...,*α*_*i*, *j*, *s*_,...,) for all *i*, *j *is performed to define a likelihood equivalent metric, called Bayesian Dirichlet equivalent (BDe) metric [21], that assigns the same score to structures entailing the same CI relationships. Given a variable  and its vector of parents , we define the number of states *r*_*i *_of *X*_*i *_and *q*_*i *_of . A value of *α*_*i*, *j*, *s *_=  for each *s *defines a likelihood equivalent metric by keeping the equivalent sample size *N *fixed to a preferred value, here equal to 9 in all our computations. In other words a virtual sample of nine observations is equally allocated to each CPT, say for a 3 times 3 table one observation is allocated to each cell in the table.

The closed-form integration of network parameters leads to:

(5)

with , and where  are sums of sufficient statistics *N*_*i*, *j*, *s*_, that is counts of cases in which the *i*^*th *^variable takes the *s*^*th*^state while its parent configuration is in the *j*^*th *^state.

Note that the marginal distribution *p*(|*ξ*) = ∑_*z *_*p*(|*z*, *ξ*)·*p*(*z*|*ξ*) becomes quickly intractable for an increasing number of nodes, and in this case the posterior distribution *p*(*z*|, *ξ*) is not available in closed form. Model selection may be performed by scoring structures and maximizing *p*(, *z*|*ξ*) with respect to *z*, both to select the best structure and to identify a restricted collection of structures on which approximated computation of posterior probabilities may be focused [21].

### MultiDAGs

A DAG captures a quite strong form of conditional independence relation because it must hold for each value taken by the conditioning variables. A weaker conditional independence relation is obtained by allowing the independence among variables to hold only for a subset of all possible states taken by conditioning variables. The new relation is sometimes called context-specific conditional independence relation.

A multi-DAG, also called Bayesian multinet [22,23], with distinguished random variable *X*_*c*_, *c *∈ *V*, is a set of component DAG models for *X*_*V*\*c*_, each encoding a joint distribution that may differ for each value *x*_*c *_taken by the distinguished variable. The extended factorization takes the following form:

(6)

with *π*_*c *_the marginal probability of *X*_*c *_= *x*_*c *_and *pa*(*v*_*i*_, *c*) the set of parents of node *v*_*i *_in the DAG component defined by *x*_*c*_.

Structural learning of multiDAGs is performed under the BDe score by iterating the search algorithm over groups of observations carrying the same value *x*_*c *_of the distinguished variable.

## Competing interests

The authors declare that they have no competing interests.

## Authors' contributions

The authors equally contributed to this work.
